# A self-cleaning underwater superoleophobic mesh for oil-water separation

**DOI:** 10.1038/srep02326

**Published:** 2013-07-31

**Authors:** Lianbin Zhang, Yujiang Zhong, Dongkyu Cha, Peng Wang

**Affiliations:** 1 Biological and Environmental Sciences and Engineering Division, Water Desalination and Reuse Center, King Abdullah University of Science and Technology, Thuwal 23955–6900, Saudi Arabia; 2 Advanced Nanofabrication Imaging and Characterization Core Lab, King Abdullah University of Science and Technology, Thuwal 23955-6900, Saudi Arabia

## Abstract

Oil–water separation has recently become a global challenging task because of the frequent occurrence of oil spill accidents due to the offshore oil production and transportation, and there is an increasing demand for the development of effective and inexpensive approaches for the cleaning-up of the oily pollution in water system. In this study, a self-cleaning underwater superoleophobic mesh that can be used for oil-water separation is prepared by the layer-by-layer (LbL) assembly of sodium silicate and TiO_2_ nanoparticles on the stainless steel mesh. The integration of the self-cleaning property into the all-inorganic separation mesh by using TiO_2_ enables the convenient removal of the contaminants by ultraviolet (UV) illumination, and allows for the facile recovery of the separation ability of the contaminated mesh, making it promising for practial oil-water separation applications.

Advanced materials with desirable wettability are very important in oil/water related applications, such as oily industrial wastewater treatment and oil spill cleanup^1^,^2^,^3^,^4^,^5^,^6^,^7^,^8^,^9^,^10^,^11^,^12^,^13^. Recently, inspired by the remarkably oil-repellent characteristic of fish scales in aqueous media, the membranes with hydrophilic and underwater superoleophobic properties have been developed and shown to be promising materials for oil and water separation^14^,^15^,^16^,^17^,^18^,^19^,^20^,^21^,^22^. These water-loving and water-permeable separation membranes are advantageous over traditional hydrophobic and oleophilic materials for two reasons: (1) they allow water to pass, which effectively avoids or reduces the possibility of membrane clogging by the viscous oil; (2) they prevent the formation of the water barrier between the membranes and the oil phase, which would otherwise occur with the conventional hydrophobic and oleophilic separation materials due to the fact that water is generally heavier than oil phase and it thus prevents the contact between oil and separation membranes.

The preparation of the hydrophilic and underwater superoleophobic separation membranes are based on hydrophilic surface modification of base materials along with generation of surface micro-nano structures, which contributes to surface roughness and amplifies surface wetting behaviors^17^,^18^,^19^,^20^,^21^,^22^. The current methods for surface hydrophilic modification can be categorized into two groups: (1) hydrophilic organic polymer-based grafting^17^,^18^,^19^,^20^ and (2) inorganic material coating^21^,^22^. Generally speaking, the inorganic coating is more desirable than the polymeric one because the latter usually suffers from poor stability and becomes unstable under harsh conditions which occur during the separation process. However, for the current inorganic coating methods, hydrothermal-based synthesis procedure is generally required^21^,^22^, which prohibits large-scale production and thus not suitable for practical applications. Furthermore, in practical applications the hydrophilic or superhydrophilic surfaces of the separation materials are prone to contamination by low-surface-energy substances due to their intrinsically high surface energy^9^,^23^,^24^. These low-surface-energy contaminants, once adsorbed, are difficult to remove and often cause the surface wetting behaviors to deteriorate, leading to the materials to lose their separation performance. It is for this reason that frequent washing-based maintenance to the separation membranes is generally required, which significantly increases the operation cost of these separation processes. It should be noted that very recently, Feng and coworkers reported a hydrothermal preparation of a double-layer TiO_2_-based mesh membrane with superhydrophobicity and superoleophilicity, which could be used for both the separation of bulk oil from water and the degradation of dissolved pollutants in water^25^. However, their work did not target the contamination problem of the separation mesh. Based on these, a facile and low-cost approach for preparation of inorganic-coating-based oil-water separation membranes, which have self-cleaning capability, is highly desired.

Layer-by-layer (LbL) assembly, which involves alternate deposition of species (building blocks) with complementary interactions to prepare composite coatings, is a versatile platform for fabricating various kinds of coatings with well-tailored chemical compositions and architectures on almost any substrate^26^,^27^,^28^,^29^,^30^,^31^,^32^,^33^,^34^. We believe LbL assembly holds a great potential for functionalizing base materials towards solving the aforementioned problems that occur with the previous oil-water separation materials based on the following considerations: (1) With LbL assembly, building block species are deposited in a rationally predesigned fashion, which enables judicially targeted functionalities and even multi-functionalities to be precisely integrated in a single coating and thus holds promise of imparting self-cleaning and/or anti-fouling functionalities into thus-prepared oil-water separating membranes. (2) The LbL assembly enables easy adjustment of the surface micro-nano structure of the coating, which is required for special wetting behaviors. (3) The LbL technique allows for the large-scale deposition of functional coatings on non-flat substrates with irregular and complicated morphology, which makes it scalable, versatile, and thus low-cost for oil-water separation applications.

Herein, we for the first time demonstrate the ease and utility of LbL assembly for the preparation of all-inorganic-coating-based oil-water separation materials. A proof-of-concept is provided by LbL assembly of sodium silicate and TiO_2_ nanoparticles on a stainless steel mesh to fabricate an underwater superoleophobic separation membrane with self-cleaning ability. The thus-prepared mesh could effectively separate water from oil-water mixture and the UV illumination provides convenient way to self-clean the contaminated mesh. The current study contributes to the development of advanced oil-water separation materials for practical applications.

## Results

**Figure 1:**
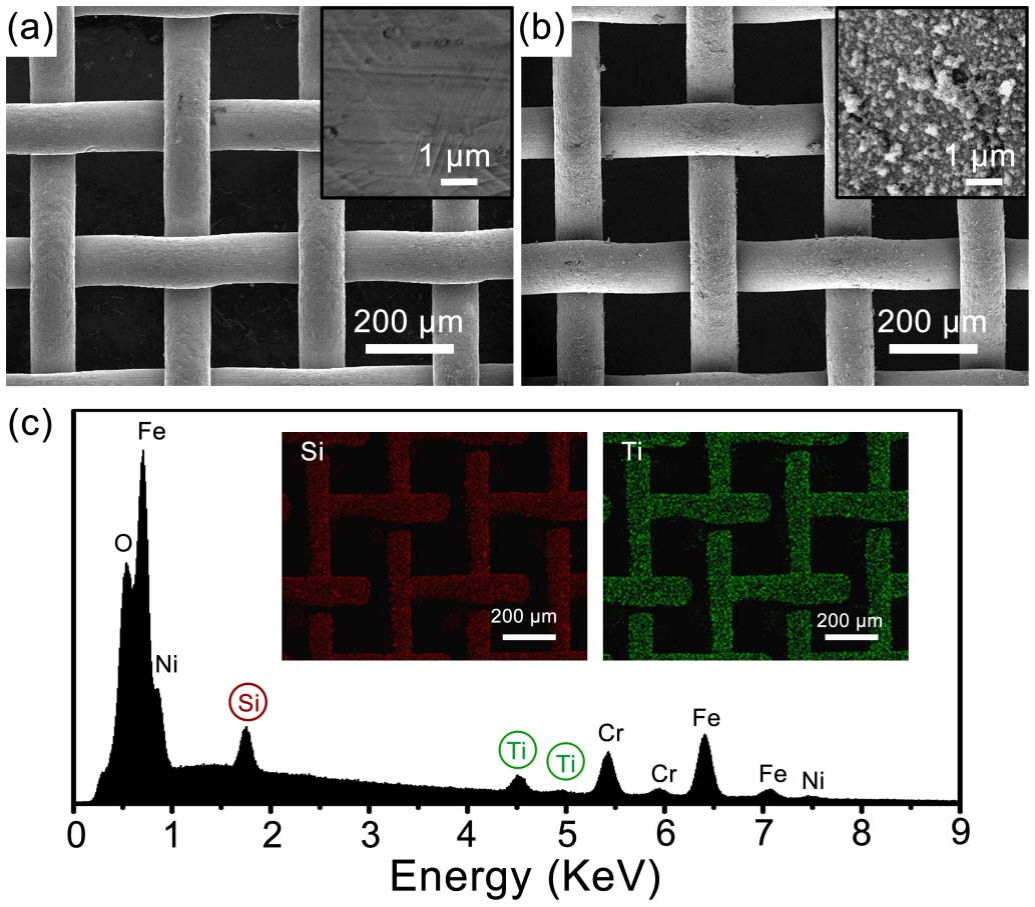
Morphology and surface composition of the stainless steel mesh after the LbL assembly of (silicate/TiO_2_)*20 coating. SEM images of the original stainless steel mesh (a), and the (silicate/TiO_2_)*20 coated mesh (b). Insets in (a) and (b) show the magnified view of the knitting wire surfaces. (c) EDS spectrum of the (silicate/TiO_2_)*20 coated mesh. The insets in (c) show the SEM-EDS elemental mapping of Si and Ti.

The choice of the steel mesh here as the base material is due to its inherent porous structure, which is suitable for separation applications, its good mechanical and chemical stability, as well as its easy availability and low cost. The self-cleaning property is imparted into the mesh-membrane by using photocatalytic TiO_2_ as one of the LbL building blocks, which provides a convenient solution to the contamination problem^35^,^36^,^37^. After 20 cycles of LbL assembly of sodium silicate and TiO_2_, which is driven by electrostatic interaction between the negatively charged sodium silicate and positively charged TiO_2_, a nanostructured composite coating of hydrophilic silica and self-cleaning TiO_2_ (denoted as (silicate/TiO_2_)*20), was formed uniformly on the surface of the stainless steel mesh. The LbL assembly of sodium silicate and TiO_2_ in this work is conducted by a non-drying LbL assembly process, in which no intermittent drying steps are involved during the deposition procedure^38^. As has been reported, the deposition of the rigid building blocks of sodium silicate and TiO_2_ onto the substrates is in their aggregated forms, and the non-drying LbL assembly process preserves the aggregates on the surface, leading to the loose stack of the building blocks and thus producing the nanostructured coating^38^, which along with micro-scale mesh wires is essential for the extreme wetting behaviors. Figure 1 shows the scanning electronic microscopy (SEM) images of the original stainless steel mesh and the one after the LbL assembly of (silicate/TiO_2_)*20 coating. The original mesh has an average pore diameter of ~ 190 μm, and the knitting wires have a diameter of ~ 110 μm. The magnified view in the inset of Fig. 1a reveals that the original wires have smooth surface. After the LbL assembly of (silicate/TiO_2_)*20 coating, the macroscopic morphology of the mesh did not show any significant change (Fig. 1b), and a layer of nano-aggregates with the size from several tens to several hundred nanometers was uniformly formed on the surface of the wires (inset of Fig. 1b). The energy-dispersive X-ray spectroscopy (EDS) measurement reveals the presence of Ti and Si on the surface (Fig. 1c), and the SEM-EDS elemental mapping results indicate a uniform distribution of the Ti and Si on the surface of the wires (insets of Fig. 1c). We found that 20 cycles of the LbL assembly of sodium silicate and TiO_2_ resulted in a sufficient coverage of the nano-aggregates on the mesh surface while when fewer cycles (e.g., 10 cycles) were assembled, only island-like aggregates were discretely distributed on the wire surface, with the underneath stainless steel exposed (see Supplementary Fig. S1, and Fig. S2 online), which is undesirable.

**Figure 2:**
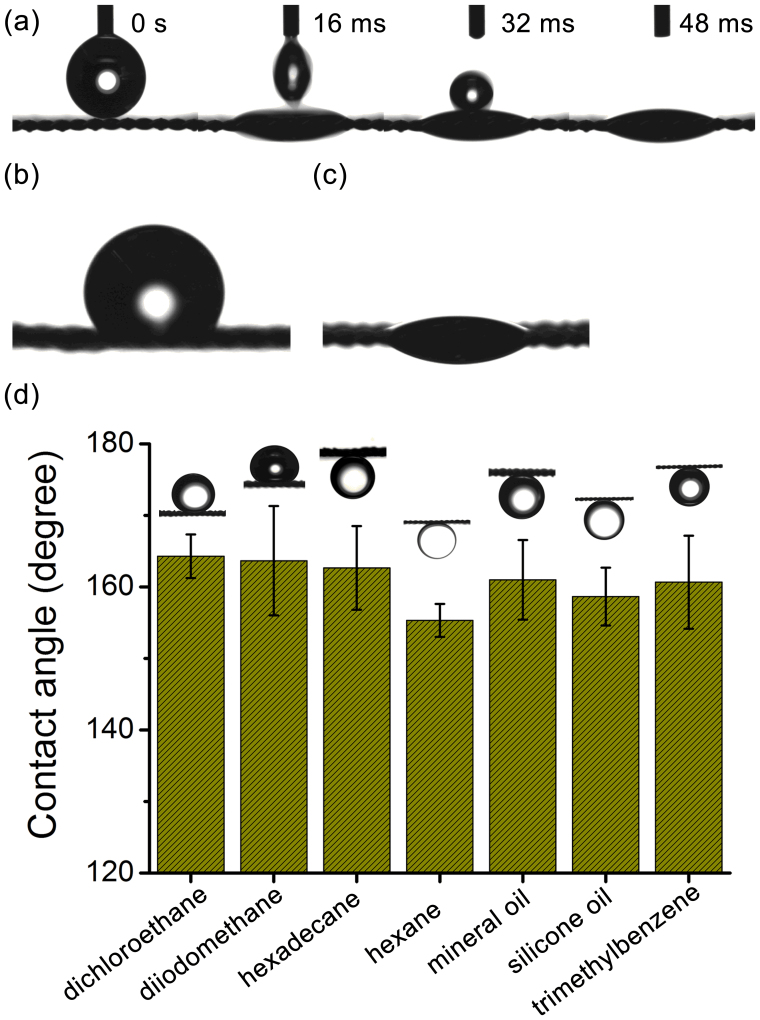
Wetting behaviors of the (silicate/TiO_2_)*20 coated stainless steel mesh. (a) Time-resolved snapshots from the contact angle measurement video of a water droplet contacting on the (silicate/TiO_2_)*20 coated mesh in air. (b) Shape of a water droplet on the original mesh. (c) Shape of a hexadecane droplet on the (silicate/TiO_2_)*20 coated mesh in air. (d) Contact angles of a series of typical oil droplets on the coated mesh in aqueous media. Insets in (d) show the shapes of the oil droplets on the mesh surface.

The wettability of water and oil on the (silicate/TiO_2_)*20 coated mesh was evaluated by the contact angle measurements. In air, the water wetting behavior on the (silicate/TiO_2_)*20 coated mesh was greatly enhanced compared with the original uncoated mesh, which had a water contact angle of 127.5°. As shown in Fig. 2a, when the water droplet contacted with the surface of the (silicate/TiO_2_)*20 coated mesh, it quickly spread and penetrated the mesh (within 16 ms), with a contact angle of ~ 21.9° above the mesh. When more water droplets were added on the mesh surface, the water could easily drip down, indicating the good hydrophilicity and permeability of the coated mesh to water, which was a combined effect of the hydrophilic nature of the LbL assembled coating and the surface micro-nano hierarchical structures generated in the non-drying LbL assembly process. The coated mesh also exhibited oleophilic property in air with a hexadecane contact angle of ~ 18.9° above the mesh surface (Fig. 2c). Meanwhile, the current LbL assembly method enables the silicate/TiO_2_ coatings to be readily deposited on stainless steel meshes with different sizes, and after coating these meshes all exhibited good hydrophilicity and water permeable property (see Supplementary Fig. S3 online).

The underwater oil wettability of the mesh was evaluated by immersing the (silicate/TiO_2_)*20 coated mesh in aqueous media. Figure 2d shows the contact angles of a series of typical oil droplets on the coated mesh in aqueous media, and the shapes of these oil droplets were also presented as the insets. Without any exception, all of the oil contact angles were larger than 150° on the coated mesh, confirming its underwater superoleophobic property. Without the coating, the original stainless steel mesh exhibited oleophilic property (see Supplementary Fig. S4 online). Furthermore, we found that these oil droplets were quite unstable on the (silicate/TiO_2_)*20 coated mesh surface and they could easily detach from the surface by gentle disturbance, suggesting a low adhesion of the surface to the oil droplets in the aqueous medium. As previously shown, the (silicate/TiO_2_)*20 coated mesh exhibited a micro-nano hierarchical surface structure and hydrophilic nature, so water could be trapped in these micro-nano structures when the mesh was immersed in aqueous medium. Because of the high repellency between polar mesh surface (water and silica) and non-polar oil phase, the mesh surface exhibits oleophobicity, which is further amplified by the surface micro-nano hierarchical structures (i.e., surface roughness), leading to an underwater superoleophobic surface^14^,^15^,^16^,^17^,^18^,^19^,^20^,^21^,^22^.

**Figure 3:**
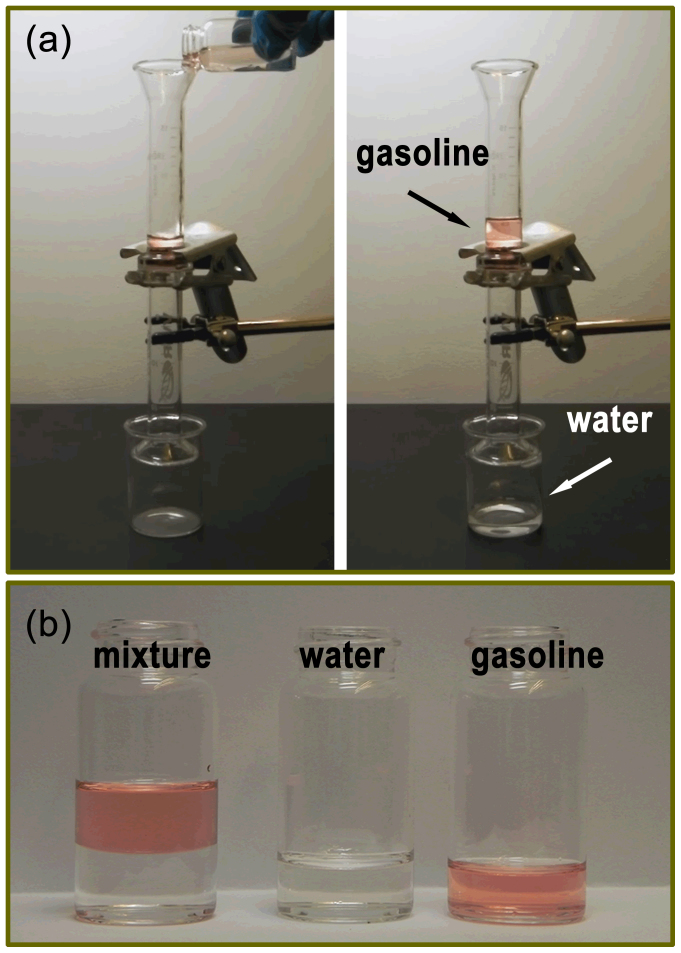
oil-water separation by the (silicate/TiO_2_)*20 coated stainless steel mesh. (a) Oil-water separation by the (silicate/TiO_2_)*20 coated mesh. The mesh was pre-wetted by water and fixed between two glass tubes as the separation membrane. A mixture of gasoline and water was poured into the upper glass tube. The water passed through the membrane, whereas the gasoline remained on top of the membrane in the upper glass tube (right panel). (b) Photograph shows collected water and gasoline after the separation. No visible oil and water was observed in the collected water and oil, respectively, indicating a complete separation.

Having demonstrated the underwater superoleophobicity, as well as the hydrophilicity and the water permeable properties of the coated mesh, the coated mesh was then used for the separation of oil and water mixture. As shown in Fig. 3a, the stainless steel mesh with the (silicate/TiO_2_)*20 coating was fixed between two glass tubes, and then a mixture of commercial No.95 gasoline and water (1:1, v:v) was poured into the upper glass tube (see Supplementary Movie S1 online). Because of the underwater superoleophobic property and the higher density of water than gasoline, the water in the mixture passed through the mesh quickly, and no visible oil was observed in the collected water. As shown in Fig. 3b, a complete separation was achieved for the oil-water mixture. Gravity was the only available force and no other external force was used during the separation.

## Discussion

**Figure 4:**
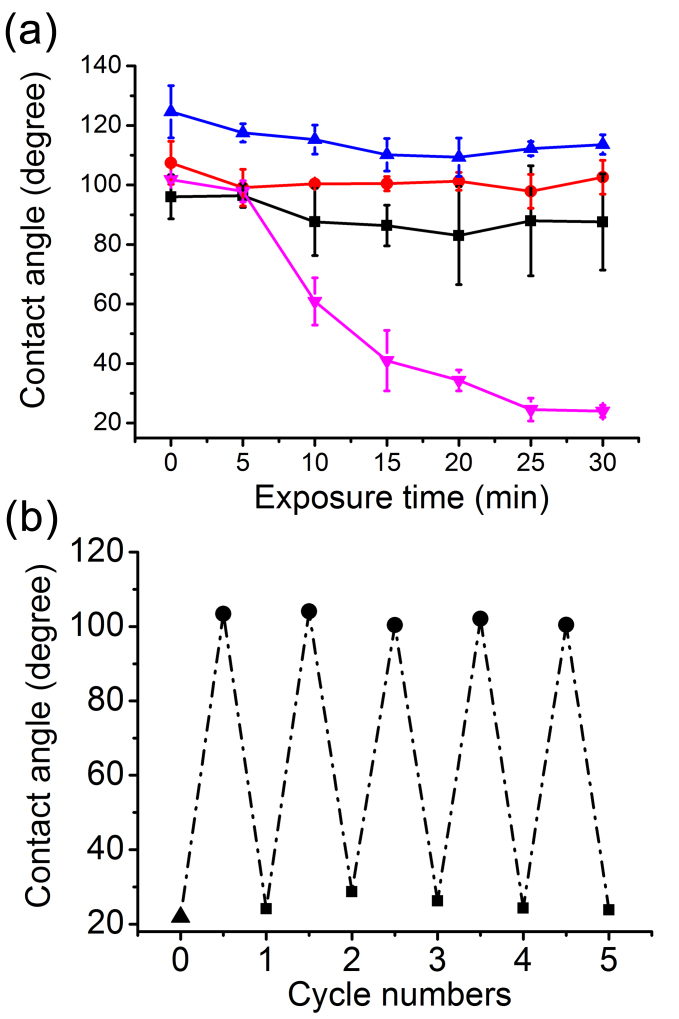
self-cleaning ability of the (silicate/TiO_2_)*20 coated stainless steel mesh. (a) Change of water contact angle of the oleic-acid-contaminated meshes as a function of UV illumination time. (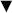) (silicate/TiO_2_)*20 coated mesh; (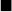) PDDA/silicate coated mesh; (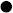) original mesh. (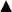) original mesh without oleic acid contamination. (b) Water contact angle changes on the (silicate/TiO_2_)*20 coated mesh in the five cycles of the oleic acid contamination and UV illumination based recovery. (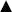) (silicate/TiO_2_)*20 coated mesh; (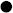) after oleic acid contamination; (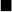) after UV illumination recovery.

As discussed earlier, self-cleaning property of a separation mesh is very desirable. It is well known that under ultraviolet (UV) light illumination TiO_2_ materials can generate photo-electrons and holes, which then react with oxygen and water to produce highly reactive species of superoxide anions and hydroxyl radicals^35^,^36^,^37^. The highly reactive species can then decompose and thus remove organic contaminants and fouling species that are absorbed on the surface of separation membrane. In order to evaluate the self-cleaning capability of the silicate/TiO_2_ coated mesh, the mesh was first contaminated with a model contaminant of oleic acid. Once contaminated by oleic acid, the surface of the (silicate/TiO_2_)*20 coated mesh lost its hydrophilicity and the underwater superoleophobic property, and showed a hydrophobic property with a water contact angle of ~ 105° in air, which undesirably led to its inability to separate oil and water (see Supplementary Fig. S5, and Fig. S6 online). With the oleic acid contamination, both water and oil passed through the (silicate/TiO_2_)*20 coated mesh indiscriminately. The contaminated mesh was then subject to UV light illumination (wavelengths centered at 360 nm) and under UV exposure for about 30 minutes, the mesh restored its hydrophilicity, with water contact angle returning to its original value of ~ 20° (Fig. 4a). The recovery of the mesh's hydrophilicity in air was a result of the removal of the surface-absorbed oleic acid.

As a comparison, the oleic-acid-contaminated (1) original mesh (i.e., unmodified with the LbL assembled coating) and (2) mesh coated with hydrophilic PDDA/silicate multilayer without TiO_2_ (see Supplementary experimental details online), both did not show any significant change in their water contact angles (larger than 90°) before and after the UV exposure (see Supplementary Fig. S7 online). These results demonstrate that the self-cleaning property of the (silicate/TiO_2_)*20 coated mesh is indeed from the TiO_2_ component in the LbL assembled coating. Furthermore, after five cycles of oleic acid contamination and UV illumination recovery, the (silicate/TiO_2_)*20 coated mesh still exhibited hydrophilic property as well as the underwater superoleophobicity similar to the uncontaminated (silicate/TiO_2_)*20 mesh (Fig. 4b, and Supplementary Fig. S8 online), indicating the good reproducibility and stability of the silicate/TiO_2_ composite coating. With the UV-illumination based self-cleaning, the mesh could be used over again for the same oil-water separation with the same performance as before the contamination. The fact that the LbL assembled silicate/TiO_2_ coating is entirely inorganic guarantees the integrity of the coating during the UV-based self-cleaning treatment.

In conclusion, we showed an underwater superoleophobic mesh with the self-cleaning ability could be readily prepared by a facile LbL assembly of sodium silicate and TiO_2_ nanoparticles on stainless steel mesh. The thus-prepared mesh could effectively separate water from the oil-water mixture and the UV illumination is a convenient approach to the self-cleaning of the contaminated separation mesh. The current study contributes to the development of advanced oil-water separation materials for practical applications.

## Methods

### Materials and chemicals

Stainless steel mesh (80 mesh) was purchased from Alfa Aesar. Sodium silicate solution, titanium isopropoxide, poly(diallyldimethylammonium chloride) (PDDA, 20 wt%, Mw ca. 100 000−200 000), silicone oil, 1,2-dichloroethane, diiodomethane, hexadecane, hexane, 1,3,5-trimethylbenzene, oleic acid, and mineral oil were all purchased from Sigma-Aldrich and used as received. Water purified in a Milli-Q (MilliPore) system was used during all the experiments. The colloidal TiO_2_ suspension was prepared by the controlled hydrolysis of titanium isopropoxide according to a previous reported method^39^. No. 95 gasoline was purchased from a local gas station.

### Fabrication of silicate/TiO_2_ coatings on stainless steel mesh

The LbL assembly of silicate/TiO_2_ coatings on the stainless steel meshes was conducted automatically by a programmable dipping robot (Dipping Robot DR-3, Riegler&Kirstein GmbH) at room temperature. A pre-cleaned stainless steel mesh was first immersed in aqueous PDDA solution (1.0 mg mL^−1^) for 20 min to render its surface positively charged, followed by rinsing with water and drying with N_2_ flow. Then sodium silicate and TiO_2_ were alternately deposited on the PDDA-modified mesh surface^38^. The mesh was first immersed in a solution of sodium silicate (0.15 M, pH = 11.6) for 10 min, followed by rinsing in three water baths for 1 min each. Then the mesh was immediately transferred to and stay in a TiO_2_ colloidal suspension (1.3 mg mL^−1^ pH = 2.5) for 10 min, followed by rinsing in three water baths for 1 min each. By repeating the above deposition process in a cyclic fashion, silicate/TiO_2_ composite coating was prepared. The assembly of silicate and TiO_2_ was repeated until the desired cycle number was reached. No drying step was used in the deposition procedure unless it was in the last layer. The LbL assembled silicate/TiO_2_ coatings with n cycle deposition are denoted as (silicate/TiO_2_)*n.

## Author Contributions

L.B.Z. and P.W. conceived the project and planned the experiments. L.B.Z. and Y.J.Z. prepared the samples and carried out most of the experiments (wettability measurements, oil-water separation, and self-cleaning). D.K.C. carried out the SEM and SEM-EDS elemental mapping measurements. L.B.Z. and P.W. analyzed the data and wrote the paper. All authors discussed the results and commented on the paper.

## Supplementary Material

Supplementary Information Supplementary Information

Supplementary Information Movie S1
