# Supplementary material for: A self-cleaning underwater superoleophobic mesh for oil-water separation
Source: Sci Rep. 2013 Jul 31;3:2326. doi: 10.1038/srep02326 (PMC3728594; doi:10.1038/srep02326)
Supplement: Supplementary Information [file srep02326-s1.pdf]

Supplementary Information for

A self-cleaning underwater superoleophobic mesh for oil-water separation

Lianbin Zhang[1], Yujiang Zhong[1], Dongkyu Cha[2] & Peng Wang[1]

[1]Biological and Environmental Sciences and Engineering, Water Desalination and Reuse Center, King Abdullah University of Science and Technology, Thuwal 23955–6900, Saudi Arabia.
[2]Advanced Nanofabrication Imaging and Characterization Core Lab, King Abdullah University of Science and Technology, Thuwal 23955-6900, Saudi Arabia.

Correspondence and requests for materials should be addressed to P.W. (peng.wang@kaust.edu.sa)

This file includes Experimental details, Supplementary Fig. S1-S8, Legend for Supplementary Information Multimedia Movie S1.

Preparation of the hydrophilic PDDA/silicate multilayer coating on the stainless steel mesh: The LbL assembly of the PDDA/silicate *multilayer* coating was conducted automatically using the programmable dipping machine as described in the paper. A pre-cleaned stainless steel mesh was first immersed in an aqueous solution of PDDA (1.0 mg mL^{-1}, pH = 6.5) for 10 min, followed by rinsing in three water baths for 1 min each. The mesh was then immediately transferred to and stay in a sodium silicate solution (0.15 M, pH = 11.6) for 10 min, followed by rinsing in three water baths for 1 min each. By repeating the above assembly process in a cyclic fashion, the PDDA/silicate composite coating was prepared. No drying step was used in the coating deposition procedure unless it was the last layer. In this study, a 20-cycle PDDA/silicate coating was prepared on the mesh, and a water contact angle of 36.5˚ was measured for such a surface.

Self-cleaning experiment: The stainless steel mesh with the (silicate/TiO_2)*20 coating and the control meshes (the original stainless steel mesh and stainless steel mesh with (PDDA/silicate)*20 coating) were contaminated with oleic acid by immersing these meshes into a 1.0% (v/v) acetone solution of oleic acid for 20 min. Then they were subject to UV illumination, and water contact angles in air were measured at fixed intervals to evaluate the self-cleaning ability of these samples.

Characterization*:* SEM images of the meshes were obtained by a Quanta 600 field-emission scanning electron microscope (FEI Company, USA). The SEM-EDS element mapping measurement was conducted by a Nova NanoSEM scanning electron microscope (FEI Company, USA). The contact angle measurements were performed with an Attension Theta system (KSV Instruments Ltd., Finland) at room temperature. In air, the water and oil contact angles on the meshes were measured by using 2 µL droplets as the indictors. In the aqueous media, various kinds of oil droplets with the volume of 4~ 6 µL were carefully placed onto the surface of the meshes, which were fixed in a glass container filled with water. An average contact angle value was obtained by measuring the same meshes at three different positions. The UV illumination (wavelengths centered at 365 nm) was provided by a LC8 light source (150 W, Hamamatsu Photonics, Japan).

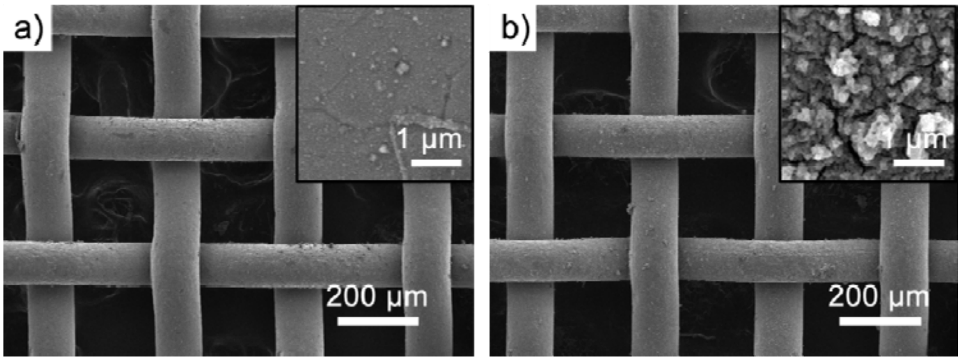

Fig. S1. SEM images of the stainless steel meshes with (silicate/TiO_2)*10 (a) and (silicate/TiO_2)*30 (b) coatings. As can be seen from ***Fig. S1a***, for the 10 cycles of sodium silicate and TiO_2 assembly, only island-like nano-aggregates were discretely distributed on the wire surfaces, representing a low coverage on the wire surface. While for the coating with the number of assembly cycles equal to and greater than 20, a uniform coverage of the wire surface by the nano-aggregates was achieved.

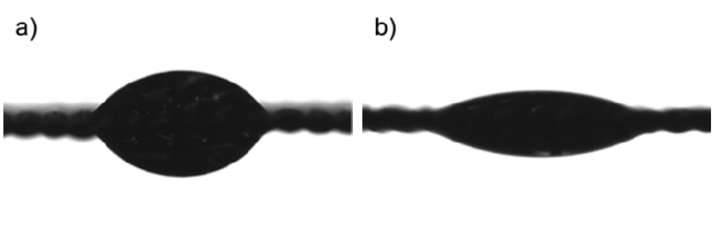

Fig. S2. Photographs of water droplets (2.0 μL) on the stainless steel meshes with (silicate/TiO_2)*10 (a) and (silicate/TiO_2)*30 (b) coating. The contact angles of these surfaces were measured to be 45.9˚ and 20.0˚ above the mesh surfaces, respectively.

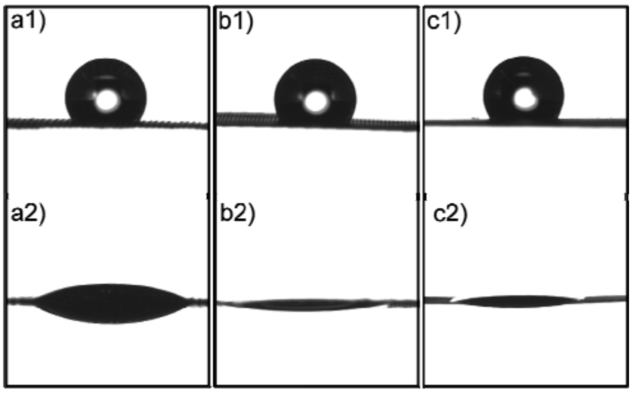

Fig.S3. Photographs of water droplets (2.0 µL) on the stainless steel meshes with different mesh sizes before (1) and after (2) the LbL assembly of the (silicate/TiO_2)*20 coatings. The sizes of these meshes are (a) 200, (b) 325, and (c) 400 mesh, respectively. For the 200 mesh (a), the wire diameter is about 48.7 µm, and the pore size is about 75.5 µm. For the 325 mesh (b), the wire diameter is about 34.6 µm, and the pore size is about 46.9 µm. For the 400 mesh (c), the wire diameter is about 28.9 µm, and the pore size is about 35.6 µm. As can be seen from these contact angles results, these original meshes all exhibited hydrophobic property in air, with the contact angles all higher than 110˚. After the LbL assembly of the (silicate/TiO_2)*20 coating on these meshes, the water contact angles on these coated meshes decreased dramatically. For the 200 mesh with the (silicate/TiO_2)*20 coating, the water contact angle was measured to be 22.7˚. For the 325 and 400 meshes with the coatings, the water contact angles were both lower than 5˚. These results demonstrated the successful assembly of the silicate and TiO_2 coating on these mesh surfaces. The improved hydrophilicity of the 325 and 400 meshes compared with that of the 200 and 80 meshes after the (silicate/TiO_2)*20 coating, was probably resulted from the enhanced capillary force due to the smaller pore size of these meshes, which is beneficial for the spreading of the water over the surfaces. Furthermore, all these coated meshes exhibited good water permeability, and when used for the oil-water separation, they all exhibited similar separation performance to that of the 80 mesh.

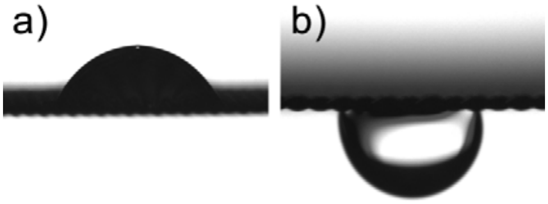

Fig. S4. The underwater oil wettability of the original mesh evaluated by using (a) dichloroethane and (b) hexadecane droplets as the indicators. These oil droplets exhibited oleophilic property on the mesh surface in aqueous media, and once contacted are difficult to detach, indicating the high adhesion of the original mesh to the oil droplets.

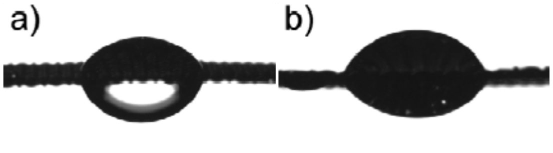

Fig. S5. The underwater oil wettability of the oleic-acid-contaminated (silicate/TiO_2)*20 coated mesh evaluated by using (a) dichloroethane and (b) hexadecane droplets as the indicators. The contact angles of dichloroethane and hexadecane on the contaminated mesh surface in the

aqueous media were measured to be 59˚ and 57.5˚ above the mesh surfaces, respectively, indicating the oleophilic property of the contaminated mesh in aqueous media.

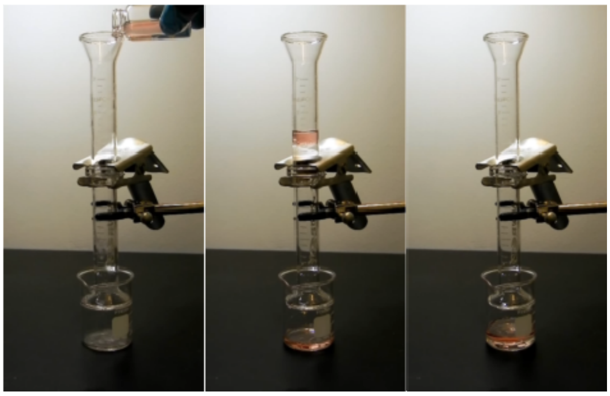

Fig. S6. Separation of oil-water mixture by an oleic-acid-contaminated (silicate/TiO_2)*20 coated mesh. Due to the loss of hydrophilicity and the underwater superoleophobicity of the contaminated mesh, the mesh failed to separate oil and water, with both the gasoline and water passing through the mesh.

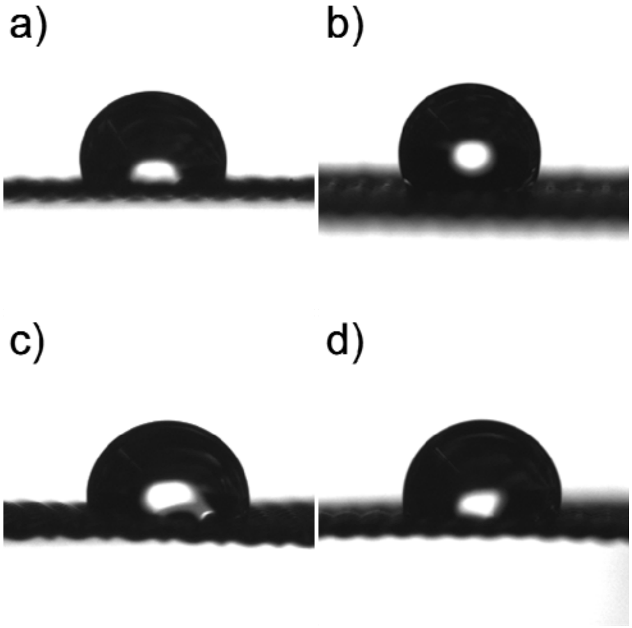

Fig. S7. (a,b) Photographs of water droplets (2.0 µL) on the oleic-acid-contaminated original stainless steel mesh (a) and after the subsequent UV illumination (b). (c,d) Photographs of water droplets (2.0 µL) on the oleic-acid-contaminated stainless steel mesh with PDDA/silicate coating (c) and after the UV illumination (d).

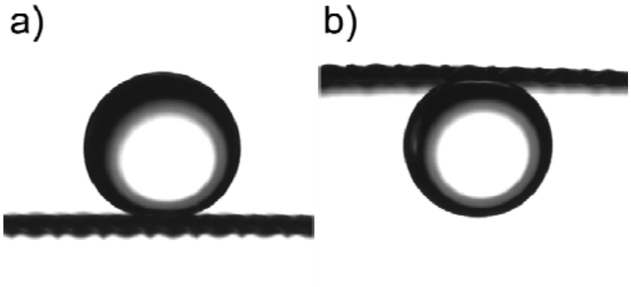

Fig. S8. The underwater oil wettability of the oleic-acid-contaminated (silicate/TiO_2)*20 mesh after the recovery by UV illumination. Dichloroethane (a) and hexadecane (b) droplets were used as the indicators, and the recovered mesh surface again exhibited underwater superoleophobic property, with the oil contact angles higher than 150˚ and low adhesion to these oil droplets.

Legend for Movie S1: Oil-water separation by the (silicate/TiO_2)*20 coated stainless steel mesh. The mesh with the size of ~ 2 × 2 cm^2 was fixed between two glass tubes (diameter ~ 1.5 cm) and pre-wetted by water, serving as a separation membrane. A mixture of commercial No. 95 gasoline and water (1:1, v:v) was poured into the upper glass tube. The water passed through the mesh membrane, while the gasoline was retained in the upper glass tube.
